# Fully Implanted Miniature Radio Controller Boosts Cyborg Insect Mobility in Challenging Terrains

**DOI:** 10.34133/cbsystems.0589

**Published:** 2026-05-25

**Authors:** Kazuki Kai, Le Duc Long, Qifeng Lin, Hirotaka Sato

**Affiliations:** School of Mechanical and Aerospace Engineering, Nanyang Technological University, Singapore 639798, Singapore.

## Abstract

Cyborg insect is a living insect equipped with electronic devices for allowing remote control of their movement. Due to their small size and locomotor capability, cyborg insects have potential advantages for application in cluttered environments where human cannot operate. Past studies have proposed various cyborg insects using different insect species and custom-made controllers equipped with sensors for desirable tasks. Those cyborg insects were usually equipped with the electronic devices on the back, leading to the loss of useful body shape of the platform organism. Although the body shape of animals is known to contribute to effective locomotion in their living environments, it has been unexamined how the arrangement of electronics to cyborg insect affects its locomotion. Here, we developed a miniature wireless controller that is fully implantable to small insects and demonstrated that the implantation of electronic device enhances traversal performance of the cyborg cockroach. The developed wireless controller was 10 mm in width, 10 mm in length, and 3 mm in height. It served sub-1 GHz communication and electrical signal output for maneuvering locomotion of cockroach. The cockroach with the implant maintained the innate tendency and traversal performance in gap negotiation comparable to intact animals, whereas the cyborg cockroach with the electronics mounted on the back exhibited degraded performance. The automatic stimulation algorithm successfully navigated the cockroach with the implant to the target with a success rate of 90.9%. The proposed technique will boost the capability of cyborg insects in challenging terrains in real scenarios.

## Introduction

Insects are often utilized as a platform for cyborgs due to their small size and locomotor capability. Acquiring sensory information from the surroundings, insects can negotiate complex, multi-component obstacles in the natural habitat that is filled with not only inorganic obstacles such as rocks and pebbles but also organic obstacles derived from living organisms, for example, wood, grass, and shrubs. In addition, insects possess remarkably high strength-to-weight ratio, meaning they can lift and carry an object heavier than their own body [1–3]. These hallmarks make insects attractive to build up cyborg animals that have potential advantages for application in complex and unpredictable environments in disaster sites.

Cyborg insects are usually equipped with electronic devices to control locomotion as it is a fundamental requirement for real application. For this purpose, researchers have investigated stimulation protocols to manipulate insect’s movement. To control the walking direction of insect, antenna stimulation via electrical current has been developed [4]. Meanwhile, stimulation to the abdomen has deployed to initiate and enhance forward motion [4]. Further investigation revealed that stimulus parameters, such as polarity [5] and temporal pattern [6], influence the effectiveness of stimulation. Targeted muscle stimulation enables control of locomotion including flight maneuver in beetles [7,8], initiation and directional control of jumping in locust [9], and galloping in beetles, a coordinated leg movement that is not observed in natural condition [10]. In these studies, custom-made printed circuit boards (PCBs) have been fabricated to implement digital-to-analog converter and wireless communication module necessary for remote control while keeping dimension small so that insect can carry.

To improve the functionality of cyborg insects, additional components have been often integrated into the PCB. For example, on-board inertial measurement unit enabled localization of the cyborg cockroach with centimeter-level accuracy [11]. Tran-Ngoc et al. [12] developed a cyborg cockroach that is equipped with an infrared (IR) camera and demonstrated on-board real-time human detection. Simple time-of-flight sensors have been adopted to assist the navigation of the cyborg cockroach by providing the distance information of obstacles to insect to escape from the corner [13,14]. Power management is also essential for cyborg insects to operate sufficient time. Biofuel cell [15,16] and a thin flexible solar cell [17] were developed to improve the power consumption of the cyborg insects. These electronics were usually mounted on the body not to disturb leg movement or body bending. Although a few studies revealed that the payload could alter the metabolic rate and walking velocity of insect [2,18], the impact of payload arrangement on traversability of cyborg insect is yet to be examined.

It is known that roundness of the insect body enhances the traversability in cluttered environment [19]. Cockroaches predominantly exhibited the roll maneuvers to traverse the gap between vertical beam obstacles. Reduction in body roundness by adding artificial shell led to decreased probability of roll maneuver and traversal performance. Experiments on deceased cockroaches and artificial robot demonstrated that a rounded body shape could enhance the traversability through the gap by reducing the resistance from environment and assisting body rotation by passive mechanical feedback. These findings suggest that the conventional arrangement of the electronics to cyborg insects (i.e., mounted on the body) may lead to degraded performance of cyborg insects in narrow space.

Much effort has been made to integrate living organism and electronics in biomedical field [20,21]. For example, the heart pacemakers have been utilized since 1958 [22]. Recent lab-on-chip systems have allowed rapid diagnostics, controlled pulsatile, and sustained delivery of drugs. As for nonhuman animals, researchers have developed wireless recording and stimulation system for rodents and avians to address neural mechanism of behavior [23–28]. Because of the advancement in the fabrication technology, such devices have been miniaturized so that they can be fully implantable into the body to improve portability and to achieve rapid and real-time application. For cyborg insects, attention has been paid for development of electrode probes to enable intimate interface between electronic and tissues [29,30]. However, there has been no study that demonstrated full implantation of electronic devices for insects.

**Fig. 1.**
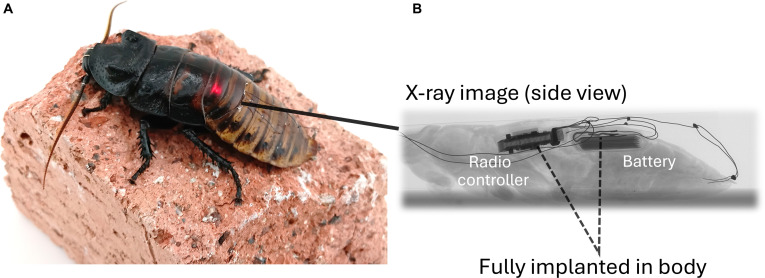
Overview of cyborg cockroach with implant. Cyborg cockroach proposed in this study (A). The LED on the controller was seen beneath the cuticle. The controller and the battery were implanted into the body (B, x-ray image).

In this paper, we constructed a fully implantable PCB for Madagascar hissing cockroach (Fig. 1). The PCB served stimulation output and wireless communication and was empowered by a small LiPo battery, which was also implantable to the insect. The cyborg cockroach with implanted PCB and battery outperformed the cyborg with conventional arrangement on the thorax. The stimulation output from the implanted PCB enabled the navigation of the cyborg in a cramped environment.

## Methods

### Animals

We used adult male Madagascar hissing cockroaches for all experiments. The cockroaches were reared in an individually ventilated cage (NexGen IVC 500, Allentown) and fed with carrots. The temperature and humidity of each cage were kept at 25 °C and 60%, respectively. To maintain the animals’ hygiene, the cages were cleaned once a week.

### Design and fabrication of the controller

First, controller form factor design was carried out to enable full implantation of the controller and the battery into the cockroach’s abdomen. Wireless communication and multi-channel electrical signal generation are finalized as 2 main block functions of the controller due to their critical impact on the study. In addition, the battery powering the controller has its size and capacity constrained due to them being implantable inside the insect body. Hence, microcontroller CC1310F128 was chosen as the main processing unit for the controller due to its small footprint of 4 mm × 4 mm and capability to perform wireless communication with ultra-low power consumption (sub-1 GHz, transmitting at 10-dBm output power/receiving current at 13.4 and 5.5 mA, respectively). Electrical signal for stimulation was generated by digital-to-analog converter (DAC) integrated circuit (IC) AD5624 with 5-V reference voltage level. Compared to other studies that either employed pulse-width modulation (PWM) signal directly from general purpose input/output (GPIO) pins of the microcontroller [6] or featured DAC AD5504 (5 mm × 6.4 mm) [12,31], not only the stimulation generator retains the robustness in signal isolation, highly accurate and customizable stimulation signal generation, but also comes with a significantly small footprint of 3 mm × 3 mm. Introducing castellated holes as debugging connector instead of standard configuration or pad connectors allows the controller to be more compact while still securing its programmable capability. The controller measured 10.0 mm long, 10.0 mm wide, and 3.5 mm high, weighing 0.5 g. A 9-mAh lithium-polymer (LiPo) battery (11.7 mm in length, 7.0 mm in width, and 3.0 mm in height) was used to empower the board.

The power consumption of the controller was measured for 60 s using EnergyTrace (Texas Instruments) via an XDS110 debugger probe (Texas Instruments). To measure the actual operation time, a controller was connected to a fully charged 9-mAh LiPo battery, and stimulation signal was generated via custom-made software every 15 s. The stimulation signal was recorded using an analog-to-digital converter (PowerLab 26T, AD Instruments), and the interval between the first and last stimulation was defined as operation time.

### Preparation of cyborg cockroach

#### Cyborg cockroach with implant

Teflon-coated silver wires (786000, AD Instruments) were soldered to stimulation channel and battery terminal on controller before insulation. Silicone elastomer (Sylgard 184, Dow) was chosen as insulation material. This material has often been used in biomedical engineering such as cell culture and the development of implantable device, due to its chemical stability, durability, and biocompatibility [32–34]. A polyethylene sheet was tightly attached inside a plastic dish. A small amount of silicone was dropped to the dish to form a thin layer and cured. Two controllers were arranged on the layer, and silicone elastomer was poured until the controllers were fully soaked. After silicone elastomer was cured, the whole block was collected from the dish by pulling the polyethylene sheet and was hand-sectioned using razor blade to isolate each controller (Fig. S1A). Batteries were insulated in the same way (Fig. S1B). A set of controller and battery implanted into cockroach was denoted as “implant” in the following sections.

A cockroach was anesthetized using carbon dioxide, and the intersegmental membrane was cut by a razor blade between the fourth and fifth or between the fifth and sixth abdominal segments, depending on its body size. The battery was pushed into the opening using a tweezer so that the stimulation channels were positioned close to the opening. The controller was implanted between the second and third abdominal segments (Fig. 1). The controller, battery, and surgical tools were sanitized using 70% ethanol before surgery.

Cockroaches use their antenna to obtain spatial information by touching object in the surrounding [35]. To preserve the function of antenna, flexible conductive fiber (Metaflex, Seiren Co. Ltd.) was used to stimulate insect’s antennae [36]. Briefly, one end of the conductive fiber was connected to the output channel of the controller through a silver wire. The other end was tied to the pedicel (second segment from the root) of the antenna. To ensure the conductivity between the fiber and antenna, conductive paste (Spectra 360, Parker Laboratories) was applied. Then, a silicone tube was used to hold the fibers on the antenna. To induce forward walking, cercal stimulation was deployed. A platinum wire (772000, AM Systems) of 10 mm length was soldered to a piece of silver wire (15 mm). The platinum wire was inserted to the cercus on each side, and the other side of the silver wire was soldered to a silver wire on the controller. As a reference electrode, a platinum wire (10 mm) was connected to a silver wire on the controller and implanted into insect body together with the controller.

#### Cyborg cockroach with backpack

For comparison, we used conventional arrangement [36], i.e., a set of controller and battery were mounted on the thorax, which is denoted as “backpack” in the following sections. The backpack had no insulation coating.

The dorsal surface of the mesothorax was roughened using a Dremel tool, and a plastic plate (10 mm × 10 mm) was secured with superglue. The controller and 9-mAh LiPo battery were arranged on the plate using double-sided tape. The height of the backpack was 4 mm including the plastic plate.

The antenna and cercal electrode were arranged as described, and the reference electrode was implanted into the third abdominal segment [36].

#### Maintenance of cyborg cockroaches

After surgery, the cockroaches were isolated in a plastic container and allowed to access food and water freely. The plastic containers were cleaned once a week to reduce the likelihood of infection and illness. Intact cockroaches that were used as the control group were kept in the same way.

### Obstacle track

**Fig. 2.**
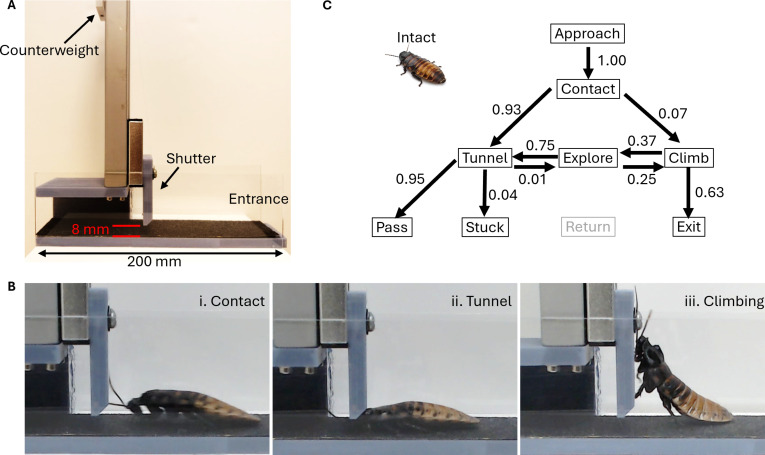
Obstacle negotiation of intact cockroach. (A) Obstacle track. The movable shutter consists of a movable stage and a 3D-printed shutter. (B) Side view of intact cockroach during (i) contact, (ii) tunnel, and (iii) climb. (C) Block diagram of intact cockroach’s behavior in the track. The number of individuals (*N*) and the number of trials (*n*) are as follows: *N* = 5, *n* = 109. Each cockroach performed up to 30 trials. Arrows represent a transition from one behavioral element to the next. The number assigned to the arrow indicates the probability of the transition.

To examine the traversal performance of the cockroaches to negotiate a narrow gap, we built a custom-made horizontal track with a movable shutter (Fig. 2A). The track measured 200.0 mm long by 47.0 mm wide and had a transparent wall (50.0 mm high and 2.0 mm thick) on each side. A linear motion guide was attached at 125.0 mm from the entrance of the track, and a 3-dimensional (3D)-printed attachment was mounted to the stage on the motion guide. A counterweight was connected to the stage with a nylon string and hung on the other side through a pulley installed on the top. The apparatus allowed precise control of the gap height and the force necessary to lift the shutter. The gap was set to 8.0 mm, and the total weight of the movable shutter was 146.5 g, whereas the counterweight weighed 96.5 g. The track was open to above for 105 mm from the entrance, and the rest was shaded by a cover to darken the track so that the cockroaches were encouraged to go under the shutter. A sheet of sandpaper (60 grit) was fixed to the track floor using double-sided tape to provide sufficient friction for the insects.

Animal’s behavior in the track was recorded using a webcam (C920, Logitech) at 30 frames/s. The resolution of the video frame was 1,920 × 1,080 pixels. The track was illuminated by a custom light-emitting diode (LED) array 1,000 mm above.

In each trial, the insect was released at the entrance of the track with the head directing toward the shutter and allowed to walk freely. If the insect failed to reach the shutter, i.e., contacting the shutter with any part of its body, it was collected and returned to the container. The insect was allowed to rest for at least 1 min before the next trial. Each trial ended when the insect (a) passed through the gap; (b) climbed over the shutter or the wall; (c) turned away from the shutter and tried to return to the entrance; or (d) spent 1 min in the track. Only such trials were accepted for analysis.

### Locomotion control and tracking

We conducted a point-to-point navigation to test the controllability of cyborg cockroaches. A custom-made software was developed to deliver stimulation to the insects based on an automatic navigation algorithm. The cyborg was released at the starting point on a flat arena and stimulated to reach the goal.

The DAC on the controller provided a biphasic square pulse train to stimulate cockroach via antenna or cerci. Amplitude was 2.5 V, pulse width was 12 ms, and the duration was 400 or 1,200 ms.

A 3D motion tracking system was used to measure the locomotion of the cockroaches during navigation. A 3D marker was attached to the thorax (implant group) or on the controller (backpack group) using double-sided tape. Six IR cameras (T40 camera, VICON) were mounted on an aluminum frame to capture a 3D marker placed on a cyborg cockroach. The software (Vicon Tracker, VICON) obtained the position and orientation of the marker. The custom-made software that was linked with the tracking software stored the locomotion data of the cyborg cockroach and stimulation parameters.

### Analysis

A chi-square test and generalized estimating equation (GEE) [37,38] were used to determine whether there are any significant differences between experiment conditions. A post hoc test was conducted for multiple comparisons with Bonferroni correction. All statistical analyses were performed on MATLAB.

All average data are given as mean ± standard error, unless otherwise mentioned. The number of individuals is denoted by *N*, whereas the number of trials is denoted by *n*.

## Results and Discussion

### Functionality of the controller

With the optimization of components selection and layout design, the controller was successfully fabricated with dimension of 10 mm by 10 mm, which is suitable for full implantation inside the insect’s body. The average power consumption of a controller during the operation was 46.74 mW. With an average current consumption of 14.16 mA, a 9-mAh LiPo battery theoretically powers up the system for 38.14 min. The actual operation time that the controller communicated with the central and output stimulation signal following the command was 32.05 min ± 1.00 min (*N* = 4). The communication distance between the controller implanted in the insect body and the central board (LAUNCHXL-CC1352R1, Texas Instruments) was 26 m.

### Gap negotiation of cyborg cockroach

We examined how intact and cyborg cockroaches react to a small gap using the obstacle track. The cockroach released in the track approached the shutter and exhibited a complex sequence of behavior to negotiate it. To quantify the entire process of the behavior, we split it into smaller elements as below.

(A) Contact: The cockroach reached the shutter and physically contacted it. Initial contact was always with antenna (Fig. 2B(i)) and could lead to either Tunnel or Climb (B and C below). This behavior included a brief stop and repetitive antennal contact to the shutter. All trial started with this phase.
(B) Tunnel: The cockroach lowered its head and contacted the shutter with the thorax (Fig. 2B(ii)). It could sweep antenna in the space behind the shutter, raise the body to lift the shutter, or walk sideways with its head down on the floor. As far as the head remained under the shutter, those behaviors were counted as a single attempt of tunneling. This phase was followed by any of Explore, Pass, or Stuck (below).
(C) Climb: The cockroach raised its body and put the leg(s) on the shutter and/or the wall but stood on the floor with any leg(s) (Fig. 2B(iii)).
(D) Explore: This was defined as a transition phase and included 2 different cases: (a) the cockroach walked backward and pulled its head out from the gap after an attempt of tunneling behavior, and (b) the cockroach descended to the floor after an attempt of climbing.
(E) Pass: The cockroach was considered to pass the shutter when the 5th abdominal segment passed the shutter because its height was smaller than the gap (8 mm).
(F) Stuck: The cockroach stopped moving under the shutter. Any part of the body between the head and the 4th abdominal segment remained under the shutter.
(G) Return: The cockroach turned more than 90 degrees away from the shutter.
(H) Exit: The cockroach completely left the floor and climbed over the shutter or the wall.

As the cockroach was given sufficient time to negotiate the obstacle, all trials ended in any of (E) Pass, (F) Stuck, (G) Return, or (H) Exit.

Intact cockroaches predominantly chose to go through the gap when they contacted the shutter (100 among 107 contacts, 93%; Fig. 2C). The insect lowered its body and pushed its head into the gap under the shutter (Fig. 2B(i) and (ii)). Because the gap was smaller (8 mm) than the insect’s body height, the cockroach was required to lift the shutter to move forward (Movie S1 and Fig. S4). Most of the intact cockroaches successfully passed through the gap (98 among 103, 95%; Fig. 2C).

**Fig. 3.**
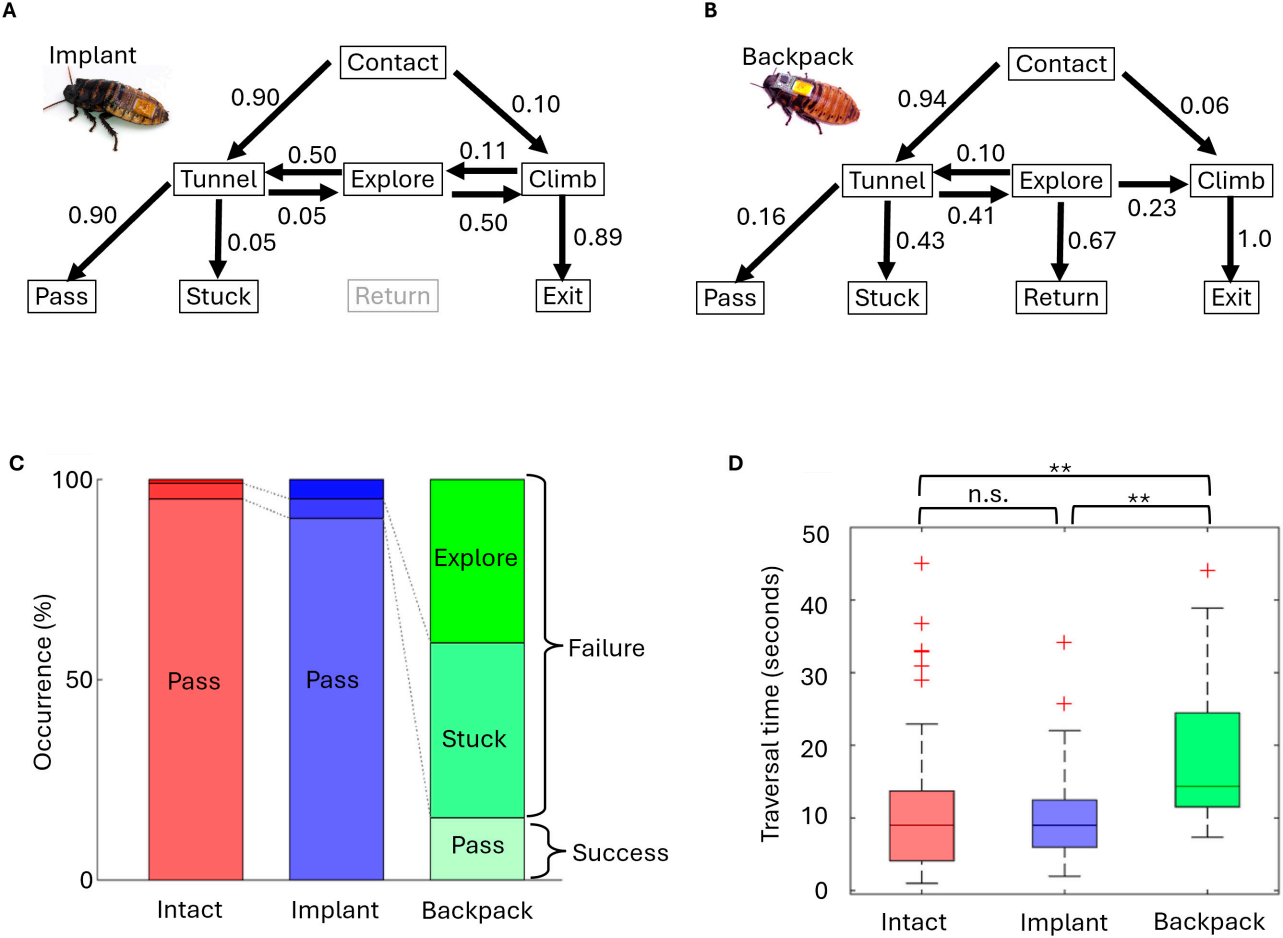
Gap negotiation of cyborg cockroach. (A) Block diagram of the behavior of cockroach with implant. The number of individuals (*N*) and the number of trials (*n*) are as follows: *N* = 5, *n* = 68. Each cockroach performed up to 16 trials. (B) Block diagram of the behavior of cockroach with backpack. The number of individuals (*N*) and the number of trials (*n*) are as follows: *N* = 5, *n* = 107. Each cockroach performed up to 26 trials. (C and D) Success rate and traversal time for Tunnel behavior. n.s., no significance. **P < 0.01. Red cross marks indicate outliers that exceed 1.5 times the interquartile value from the 75th percentile.

The cyborg cockroaches with implant exhibited the capability of gap negotiation comparable to the intact group (Fig. 3A and Movie S2). Most of this group chose to tunnel the shutter (60 of 67, 89%) when contacted it. Of all 62 attempts to tunnel, 90% (56 attempts) were successful. Half of the unsuccessful trials were followed by the Explore phase and the rest ended in the Stuck phase.

**Fig. 4.**
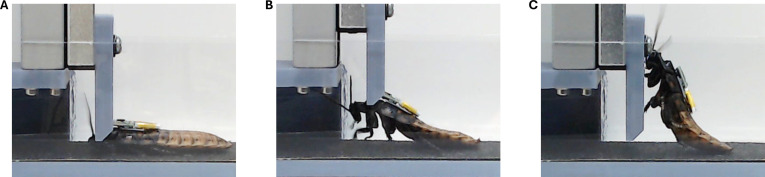
Gap negotiation of cockroach with backpack. (A) The cockroach inserted its head into the gap with the backpack hitting straight to the shutter. (B) Cockroach lifting the shutter. (C) The cockroach bent its body when climbing up the shutter.

The cyborg cockroach with backpack struggled to go into the gap. Most of cockroaches in this group tried to tunnel under the shutter after first contact (99 among 107 attempts, 94%; Fig. 3B). When the cockroach turned down its head to go under the shutter, the backpack disturbed smooth transition to tunneling. To break into the gap, the cockroach needed to lift the shutter much higher than its body height repeatedly (Fig. 4B and Movie S3). The cockroaches often gave up tunneling it and proceeded to the Explore phase followed by returning (Movie S4) or climbing (Fig. 4C). As a result, only 16 of 103 attempts (15%) of Tunnel were successful (Fig. 3B). Most tunnel attempts (45 among 103 attempts, 43%) ended in Stuck, indicating that the cockroach settled down at the shutter or failed to pass through it within 1 min. Meanwhile, no cockroach failed to climb up the shutter or wall due to the backpack (Fig. 3B).

To examine if the success rate of tunneling differed between 3 groups, we conducted a statistical test (Fig. 3C and D). If an attempt of tunneling behavior ended in pass, we counted it as success, otherwise failure. There was significant difference among groups (*P* < 0.01, χ^2^ test). A post hoc test detected significant difference between the intact and mounted group (*P* < 0.01), and between the mounted and implanted group (*P* < 0.01), whereas there was no difference between the intact and implanted group (*P =* 0.20).

We measured the time that the cockroach spent passing through the gap as traversal time (Fig. 3D). The intact cockroach spent 9.56 s ± 1.09 s (*n* = 98). The cockroach with backpack required longer time (20.60 s ± 3.16 s, *n* = 16), whereas the cockroach with implant passed with shorter time (8.10 s ± 0.70 s, *n* = 56). The traversal time was significantly different among groups (GEE, *P* < 0.01). A post hoc test detected significant difference between the intact and backpack group (*P* < 0.01), and between the backpack and implant group (*P* < 0.01), whereas there was no difference between the intact and implant group (*P =* 0.61).

These results demonstrated that cyborg cockroaches with implant maintained the innate tendency and traversal performance for gap negotiation, suggesting the potential advantages in cluttered environment.

### Controllability of cyborg cockroach with the implant

**Fig. 5.**
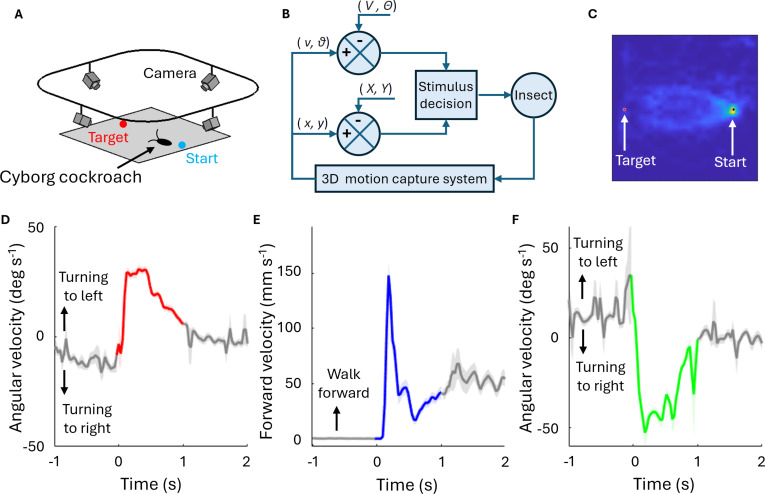
Locomotion control of cyborg cockroach. (A) Schematic view of experiment setup. Blue circle: virtual starting point. Red circle: virtual target. (B) Block diagram of automatic navigation algorithm. *X*, *x* coordinate of the target; *Y*, *y* coordinate of the target; *x*, *x* coordinate of the cyborg; *y*, *y* coordinate of the cyborg; *V*, threshold for forward velocity; *Θ*, threshold for antenna stimulation; *v*, forward velocity of the cyborg; *θ*, angular velocity of the cyborg. (C) Density map during navigation. (D to F) Angular and forward velocity of the cyborg. The thick lines indicate the mean value, and the shaded areas indicate standard error. The number of cyborgs (*N*), number of trials (*n*), and stimulations (*n*) are as follows: trials of navigation (*N* = 5, *n* = 66), forward stimulation (*N* = 5, *n* = 49), left stimulation (*N* = 5, *n* = 327), and right stimulation (*N* = 5, *n* = 210). Each cyborg performed up to 16 trials of navigation.

It is well known that the cockroach initiates walking or accelerates when electrical stimulation is delivered to cerci and shows left/right turning to right/left antenna stimulation [18,36]. Using this stimulation protocol, the cyborg cockroach was stimulated to navigate from the virtual starting point to the virtual target to examine their maneuverability (Fig. 5). The automatic navigation algorithm stimulated cerci for acceleration when the mean walking velocity of the cyborg cockroach in the last 3 s was below 5 mm/s. On the other hand, the algorithm stimulated left antenna when the heading of the cyborg was biased larger than 45 degrees to the left from the target and vice versa. During the navigation, the cyborg cockroach could spontaneously turn to left or right, and then, it received antenna stimulation to turn back to the target. As a result, our cyborgs successfully reached the target (60 of 66 trials, 90.1%). The reaction to each type of stimulation confirmed the faithful locomotion control of the cyborg cockroach (Fig. 5D to F). The mean angular velocity for left stimulation (right antenna was stimulated) was (16.65 ± 1.02) degrees/s, whereas (−26.25 ± 2.33) degrees/s for right stimulation. Because the navigation algorithm provided forward stimulation to cerci when the cyborg cockroach stopped walking, the mean walking velocity before stimulation was close to zero (Fig. 5E), and the cockroach was induced to initiate walking after stimulation. The forward velocity decreased after stimulation but remained above zero, indicating that the cyborg cockroach continued to walk forward. The mean forward velocity during 1 s after stimulation was (39.98 ± 3.04) mm/s.

### Navigation in cramped environment

**Fig. 6.**
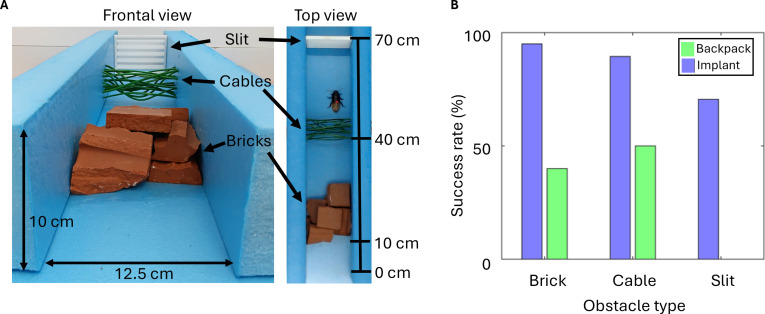
Navigation of cyborg cockroach in obstructed environment. (A) The cyborg cockroaches were released at the entrance (0 cm) of a corridor (height: 12.5 cm, width: 10 cm), which included different types of obstacles. Eight pieces of bricks were stacked at 10 cm from the entrance (0 cm) to block the corridor, 16 cables (4 mm × 2 mm) were randomly arranged within 6 cm × 6 cm area at 40 cm, and a 3D printed plastic slit with 1.5 cm gap was placed at 70 cm from the entrance. (B) Success rate for obstacle negotiation. The number of individuals (*N*) and trials (*n*) were as follows: backpack (*N* = 4, *n* = 20), implant (*N* = 4, *n* = 20).

To validate the advantage of implantation of electronics, cyborg cockroaches were navigated in an obstructed environment (Fig. 6). The cyborg cockroaches were released in a corridor with 3 types of obstacles and manually stimulated to complete the corridor within 3 min. Most cyborg cockroaches with implant (95%, 19/20) successfully traversed the bricks. The only failure mode was that the cyborg crawled into the gap between bricks and was settled. On the other hand, only 40% of backpack cyborgs passed the bricks with longer traversal time (backpack: 93.40 s ± 11.90 s and implant: 54.16 s ± 7.80 s, *P* < 0.01, GEE). The implant group achieved 89.4% success rate for negotiating the cables (17/19). The backpack group (4/8, 50%) could negotiate the cables, but the average traversal time was 3 times longer than the implant group (54.67 s ± 11.73 s and 13.65 s ± 2.37 s, *P* < 0.01, GEE) because the cyborgs with backpack were required to reroute for finding the larger space or to push up the cable repeatedly to obtain a chance to go under it. No backpack cyborgs (0/4) succeeded in going through or climbing over the slit, whereas 63.1% (12/17) of implant cyborgs could traverse it (Fig. 6B). The average traversal time for the slit was 29.08 s ± 6.68 s. Overall, the cyborg cockroach with implant outperformed the conventional cyborg with backpack in both success rate and traversal time. This result suggested that implantation of electronic devices can facilitate navigation of the cyborg cockroach in cramped environment.

### Impact of implantation

The post-implantation survivorship of cyborg cockroaches was monitored for up to 120 d. The first batch of cyborg cockroaches exhibited highly variable outcomes; 4 of 7 individuals died within 7 d, and the rest survived for more than 80 d (Table S1). For those cockroaches, the controller and the battery were forcibly inserted into the body, dislocating (or possibly tearing) the internal tissues with the implanted electronics. To seek for smooth implantation, we split up the insertion process into 2 steps for the second batch of cyborg cockroaches. After cutting the intersegmental membrane, a rounded-shaped spatula was gently inserted from the opening to detach the tissues from the cuticle, and then the electronics were implanted. As a result, 6 of 7 cockroaches (85.7%) survived longer than 50 d after implantation (Table S1). Although the survival period remained variable, a trend was observed in which the survivorship at 7 d was enhanced (*P =* 0.09, χ^2^ test). The experiments in this paper were conducted using long survivors except for one insect (Animal 1 in Table S1) in the gap negotiation experiment (see the Gap negotiation of cyborg cockroach section). However, there was no significant difference between the short and long survivors in both the traversal time (*P =* 0.43, GEE) and success rate (*P =* 0.58, χ^2^ test). Further refinement of surgical techniques, reduction of implant size, use of softer encapsulation materials, and application of anti-infective treatments are expected to improve survivorship. For both emergency short-term missions such as search and rescue (SAR) and planned non-urgent applications such as pipeline or infrastructure inspection, maintaining survivorship beyond 72 h can be considered a practical operational benchmark. In infrastructure inspection tasks, each mission typically covers a limited section bounded by manholes—generally on the order of several tens of meters—and such operations are usually completed within several hours to a day. Therefore, a 3-d survival duration is sufficient to ensure mission completion and to align with the practical replacement cycle of cyborg units. Although survivorship is not the focus of this study, these data are presented to clarify that the implanted cockroaches were able to remain viable well beyond the immediate post-operative period.

To examine whether the implantation influences insect’s locomotor activity, we have analyzed the leg movement of cyborg cockroaches (Fig. S2). Both intact and cyborg cockroaches exhibited tripod gait while walking straight. There were no significant differences in the stance–swing ratio and phase difference between intact and cyborg cockroaches, suggesting that implantation did not alter gait patterns. Similarly, the roundness of the body did not change compared before and after implantation (Fig. S3).

### Limitation and future improvement

The electronic system developed in the present study serves stimulation output and wireless communication for cyborg insect. To operate in real situation, cyborg animal requires additional functionalities such as IR sensing for human detection [12], image streaming for inspection [18], distance measurement for obstacle negotiation [13,14], acoustic monitoring [39], and inertial measurement for localization [11]. Our results suggest that a synergetic arrangement of the electronics and the platform animal will enhance the locomotor performance in complex environment.

Despite successfully demonstrating insect navigation control in cramped environment, there is room for improvement for machine–insect fusion. First, the electronic system has potential to come with even more compact sizes. This is feasible by combining all main IC (microcontroller, DAC, power management unit, passive component if applicable) into a single system-on-package (SoP). Further miniaturization will improve the arrangement of the implant inside the body to reduce physical interaction with the critical organs, such as the nerve cord and the heart, to improve the survival rate. Kakei et al. [17] demonstrated that cyborg cockroaches exhibited degraded performance for traversal of obstacles and self-righting when they lost flexibility of their abdomen due to the body-mounted film, requiring longer time to walk over the obstacle and showing low success rate in self-righting. Replacing rigid PCB material and components by flexible alternatives such as soft bioelectronic devices will allow the control system to fit with the curved form of insect abdomen cuticle; hence, the compatibility of machine and insect parts is enhanced.

Power consumption is improved by setting the device communication in non-beacon mode, where the network coordinator (central transmitter device) does not broadcast periodic beacon signal for network synchronization and the implanted devices will communicate with network coordinator using CSMA/CA (carrier sense multiple access with collision avoidance) mechanism instead. Furthermore, improvement on power system can be accomplished by replacing LiPo battery by biofuel cell [15,16] or ultrathin film solar cell [17], subjected to operation condition. Such methods reduce the need for invasive operation and lead toward a harmonic fusion of machines and insects.

**Fig. 7.**
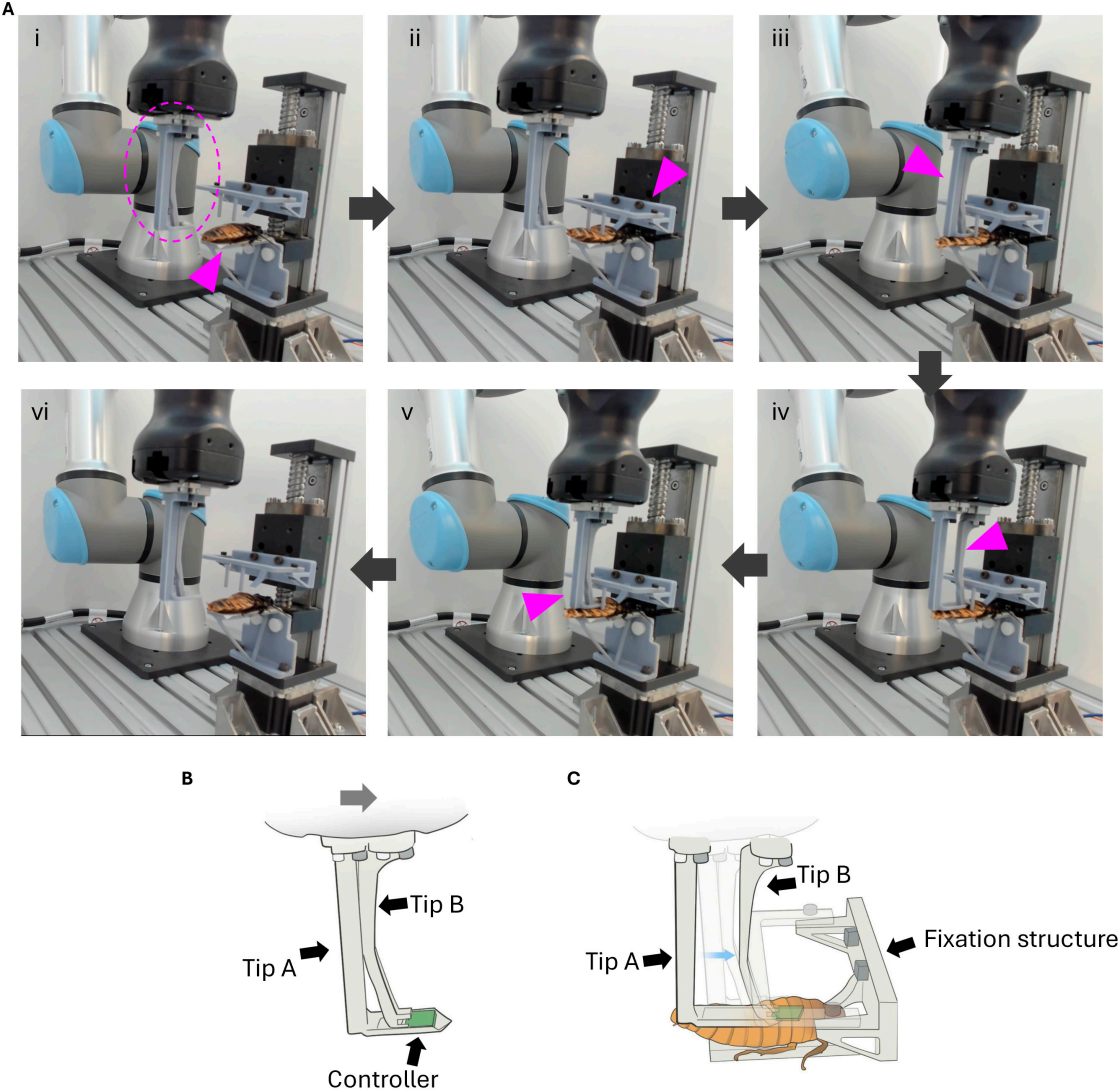
Automatic implantation of the controller. (A) Snapshot of whole process. Arrows indicate the flow of process. (i) An anesthetized insect was placed on the platform (arrowhead). The robotic arm stood by at the original position. The dashed circle indicates the fingertip of the robotic arm enlarged in (B). (ii) The fixation structure (arrowhead) on the motion guide moved down to fix the insect on the platform. The fixation structure pressed the thorax and the abdomen to make a gap between the abdominal segments. (iii) Robotic arm approached the insect. Tip A punctuated the insect’s intersegmental membrane. (iv) Tip B (arrowhead) moved toward the insect, and the controller was pushed into the insect’s abdomen [enlarged illustration in (C)]. As the dimension restricted, the controller was not fully inserted to the insect at this step. (v) Tip A (arrowhead) moved forward to push the controller further to finish implantation. (vi) Robotic arm and fixation structure retreated to the original position for the next implantation. (B) Structure of the fingertip of the robotic arm’s parallel gripper. Gray arrow indicates the forward direction to the fingertip. The controller was placed on Tip A. (C) Movement of fingertip to insert the controller into the abdomen. After Tip A punctuated the intersegmental membrane, Tip B moved forward and pushed the controller on Tip A to insert it into the abdomen (blue arrow).

As the manual preparation led to high requirements for the operator and unnecessary damage for the insects due to the operating error [40], automatic manipulation of the robotic arm to enable the implantation of the controller was considered in this study (Fig. 7 and Fig. S5). To enable the automatic insertion, a puncture structure (Tip A; Fig. 7B) was designed at one fingertip of the parallel gripper. Tip A first carried the controller and pierced the fixed insect’s intersegmental membrane. To avoid unnecessary damage of the insect’s intersegmental membrane, the width Tip A was designed at 12 mm, only 1 mm left for left and right walls for the controller. The motion of the robotic arm and its fingertip (Fig. 7A) was fully programmed, and the whole process, from fixation of the insect to the controller fully implantation and fixation structure retreated, lasted for 25 s. We conducted robotic arm implantation and found that the success rate was 100% (*N* = 5, 6.30 cm ± 0.16 cm in body length).

Recently, Lin et al. [40] have developed an automatic assembly system for cyborg Madagascar hissing cockroach using robotic arm. Integrating deep learning-based vision system and robotic arm, the system automatically identified the point-of-interest in the thorax and carried out electrode implantation for stimulation. Our system described here operated without visual guidance and achieved the success rate of 100% in middle-sized cockroaches with body length of 6 cm due to the customized design of the fixation structure and the fingertip. Application to smaller or larger animals including other species should be possible, while it requires a modification of those structures.

**Fig. 8.**
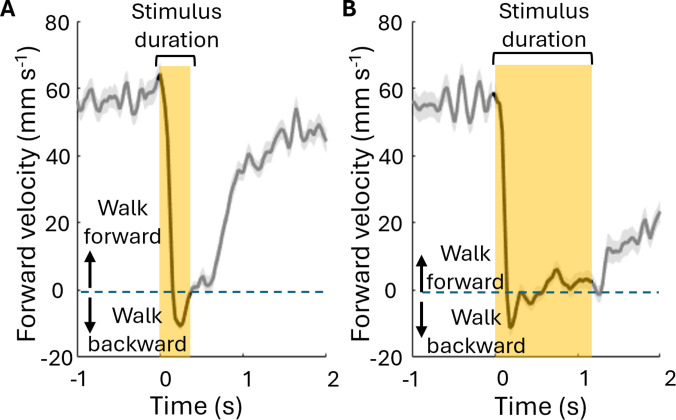
Electrical stimulation to both antennae induced backward walking. Forward velocity during 400-ms stimulation (A) and 1,200-ms stimulation (B). The gray-shaded area indicates standard error, and the yellow-shaded area represents the period when the antenna stimulation was provided. The number of cyborgs (*N*) and the number of stimulations (*n*) are as follows: 400-ms stimulation (*N* = 4, *n* = 59), 1,200-ms stimulation (*N* = 4, *n* = 49).

Cyborg cockroaches are often required to move backward when they encounter a dead end or a small gap that is too small to go through. To overcome such situations, we developed a stimulation protocol for backward walking (Fig. 8). The short stimulation (400 ms) passed through both antennae induced a reduction in forward velocity (Fig. 8A). The longer stimulation with 1,200-ms duration elicited prolonged reaction (Fig. 8B).

## Conclusion

In this work, we have developed a wireless controller for small insects and demonstrated the advantage of implantation. The cyborg cockroach with the implant showed augmented traversal performance in gap negotiation compared to the cyborg with the controller on the thorax. The remote stimulation from the implanted controller enabled locomotion control of the cyborg insect and navigation in cramped environment. There is still room for improvement for implantation protocol and survivorship of platform animals; however, this technology will boost the capability of cyborg cockroaches in cluttered environments expected in real scenarios such as collapsed buildings and narrow pipelines.

## Data Availability

The data that support our findings of this study are available from the corresponding author upon reasonable request.
