# Supplementary material for: Fully Implanted Miniature Radio Controller Boosts Cyborg Insect Mobility in Challenging Terrains
Source: Cyborg Bionic Syst. 2026 May 25;7:0589. doi: 10.34133/cbsystems.0589 (PMC13199645; doi:10.34133/cbsystems.0589)
Supplement: Supplementary 1 — Figs. S1 to S5 Table S1 Movies S1 to S4 [file cbsystems.0589.f1.zip › Supplementary Materials.docx]

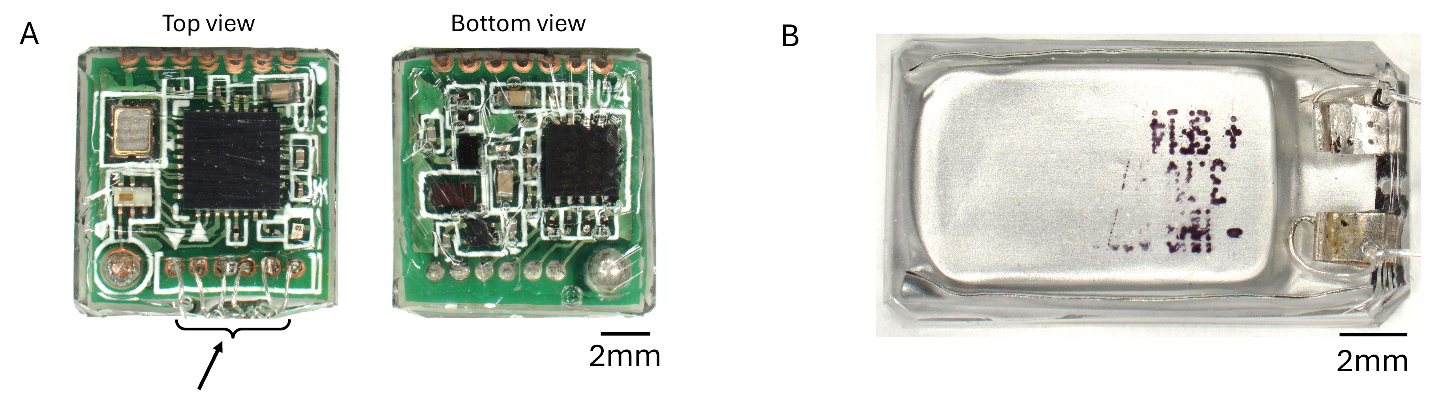


***Supplementary Fig. 1. Preparation of experimental animal. A.*** *The controller with silver wire and silicone coating. Silver wires were soldered on the top side (Arrow). Four channels on the left were used for output and two channels on the right was the battery terminal.* ***B.*** *LiPo battery coated with silicone.*


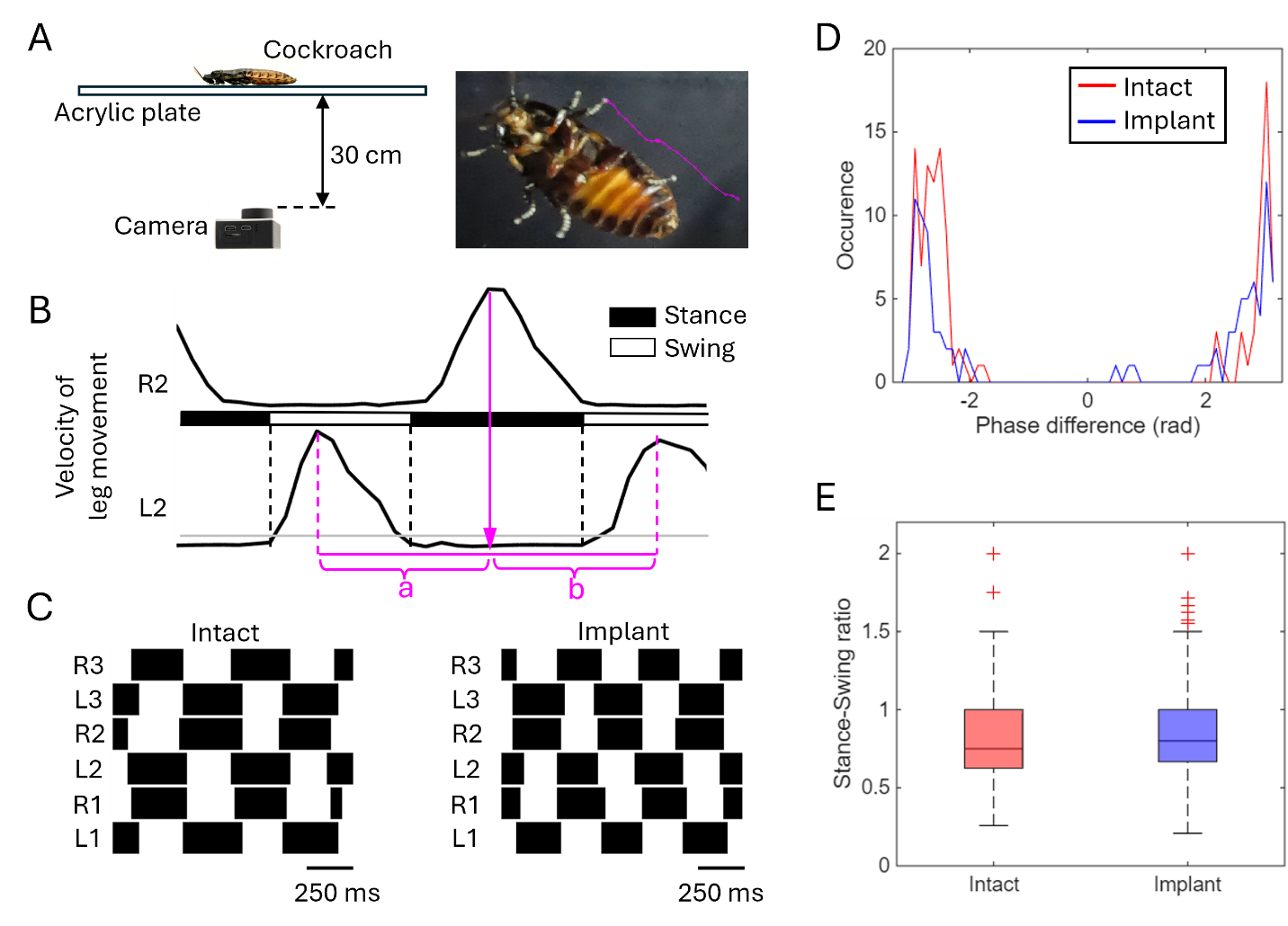


***Supplementary Fig. 2****. Comparison of walking pattern of intact and cyborg cockroach with implant.* ***A.*** *Right panel: Recording setup. A cockroach was released on a transparent acrylic plate and recorded at 50 Hz using a digital camera (GoPro 10). Right panel: Detection of movement. The position of middle legs was detected using automatic detection software (DLTdv 8).* ***B.*** *The speed of leg movement was calculated as the straight-line distance of the middle leg between the frames. The swing phase was defined as the period where the speed of left leg exceeded 3 × median(speed)/0.6745, otherwise the stance phase. The phase difference was defined as a/(a + b) and wrapped to the range from -ϖ to ϖ, where a and b were the periods between the timing of the peak of the speed of left and right middle legs. L2 and R2 indicate left and right middle leg, respectively.* ***C.*** *Gait pattern of intact and cyborg cockroach. L1/R1: left/right foreleg, L2/R2: left/right middle leg, L3/R3: left/right hindleg.* ***D.*** *Phase difference. There was no significant difference between phase difference between left and right middle leg of intact and cyborg cockroach (p = 0.21, Watson’s U2 test).* ***E.*** *Stance-swing ratio. There was no significant difference between stance-swing ratio of intact and cyborg cockroach (p = 0.83, one-way ANOVA).*


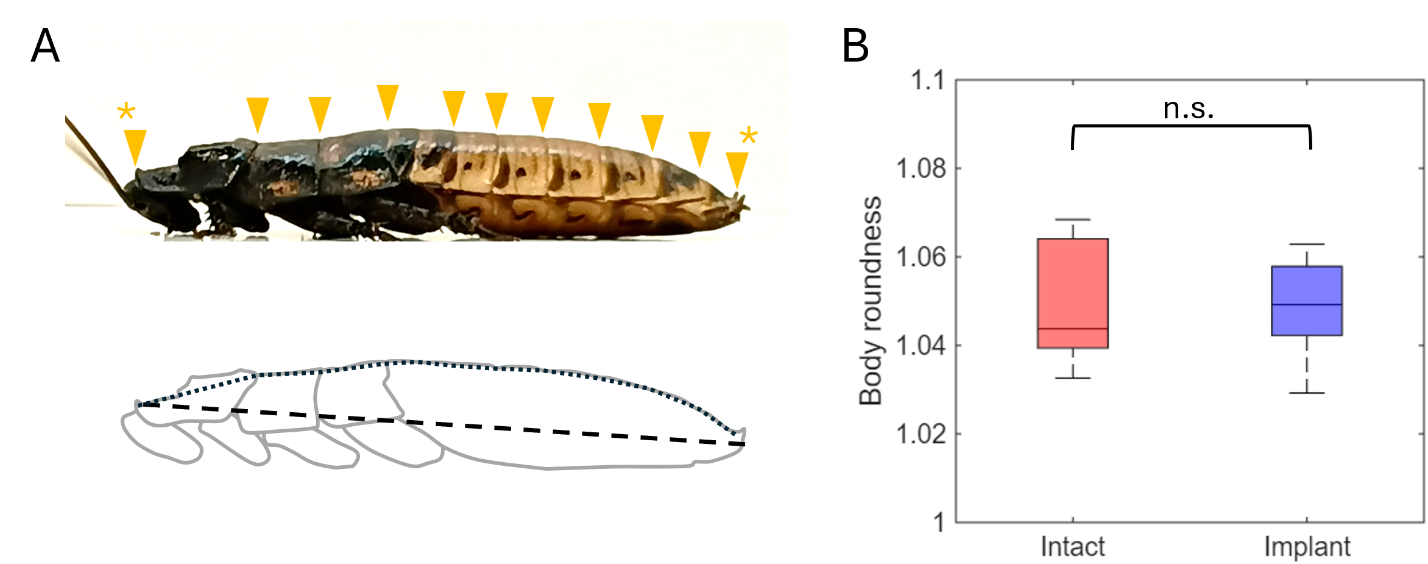


***Supplementary Fig. 3.*** *Comparison of body roundness of intact and cyborg cockroach with implant.* ***A.*** *Measurement of body roundness. Body roundness was defined as the length of the dorsal surface of the body against the linear distance from the head to the tail (upper panel, arrowheads with asterisk; lower panel, dashed line). The length of the dorsal surface of the body was defined as the polyline interconnecting the edge of body segments (upper panel, arrowheads; lower panel, dotted line).* ***B.*** *Body roundness. There was no significant difference between intact and cyborg cockroach (p = 0.97, one-way ANOVA).*


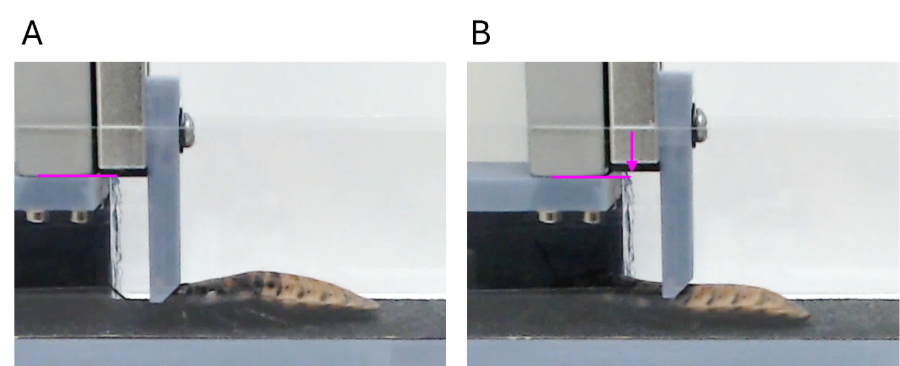


***Supplementary Fig. 4. Cockroach lifting the shutter.*** ***A.*** *cockroach in Tunnel phase.* ***B.*** *cockroach passing through the gap by lifting it. The level of horizontal magenta line in each picture indicates the same height. Note that there is a small gap between the magenta line and the bottom edge of the stage (right, arrow).*


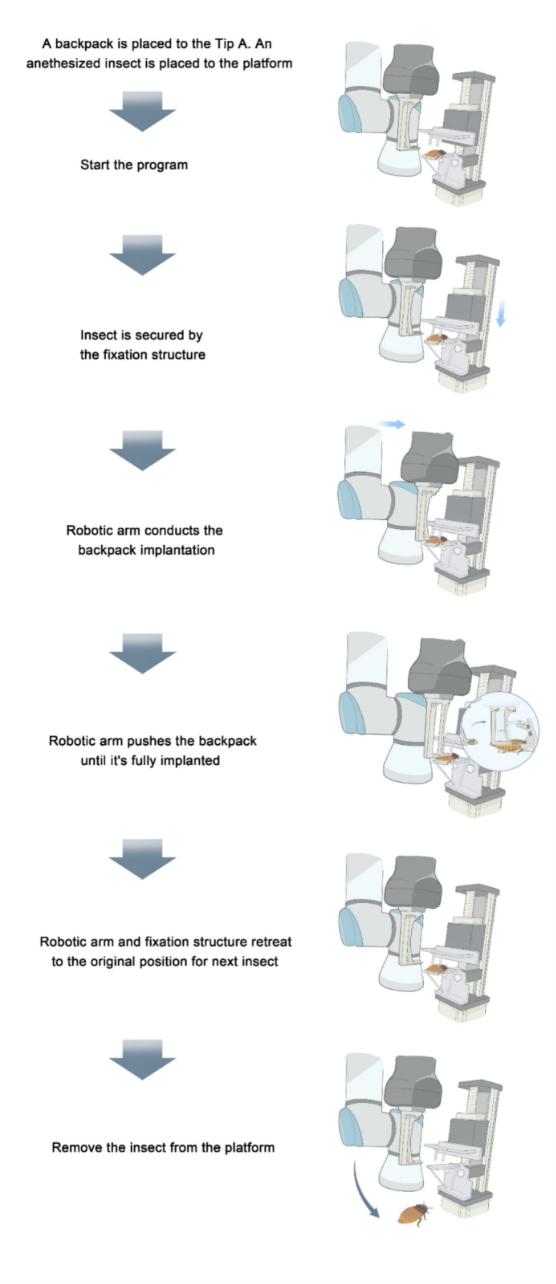


***Supplementary Fig. 5. Flowchart of automatic implantation process.***

**Supplementary Table 1**

| First batch | | Second batch | |
| --- | --- | --- | --- |
| Animal**​** | Survival period  (days)**​** | Animal | Survival period (days) |
| 1​ | 4​ | 8 | 2^#^ |
| 2​ | 5​ | 9 | 53 |
| 3​ | 6​ | 10 | 92 |
| 4​ | 7​ | 11 | 120^*^ |
| 5​ | 89​ | 12 | 120^*^ |
| 6​ | 120^*^​ | 13 | 120^*^ |
| 7​ | 120^*^​ | 14 | 120^*^ |

*Data was arranged in ascending order based on the survival period. Single asterisk (*) indicates the animal completely survived the monitoring period of 120 days.*
