# Supplementary figures and images for: Fully Implanted Miniature Radio Controller Boosts Cyborg Insect Mobility in Challenging Terrains
Source: Cyborg Bionic Syst. 2026 May 25;7:0589. doi: 10.34133/cbsystems.0589 (PMC13199645; doi:10.34133/cbsystems.0589)

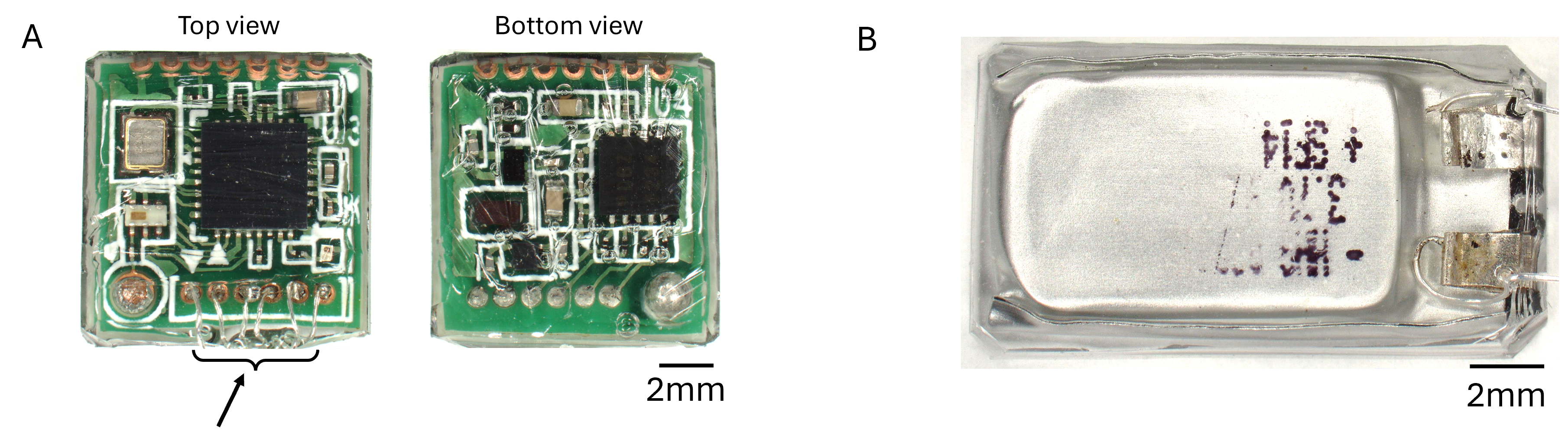

Supplement: Supplementary 1 — Figs. S1 to S5 Table S1 Movies S1 to S4 [file cbsystems.0589.f1.zip › SFig1.png]

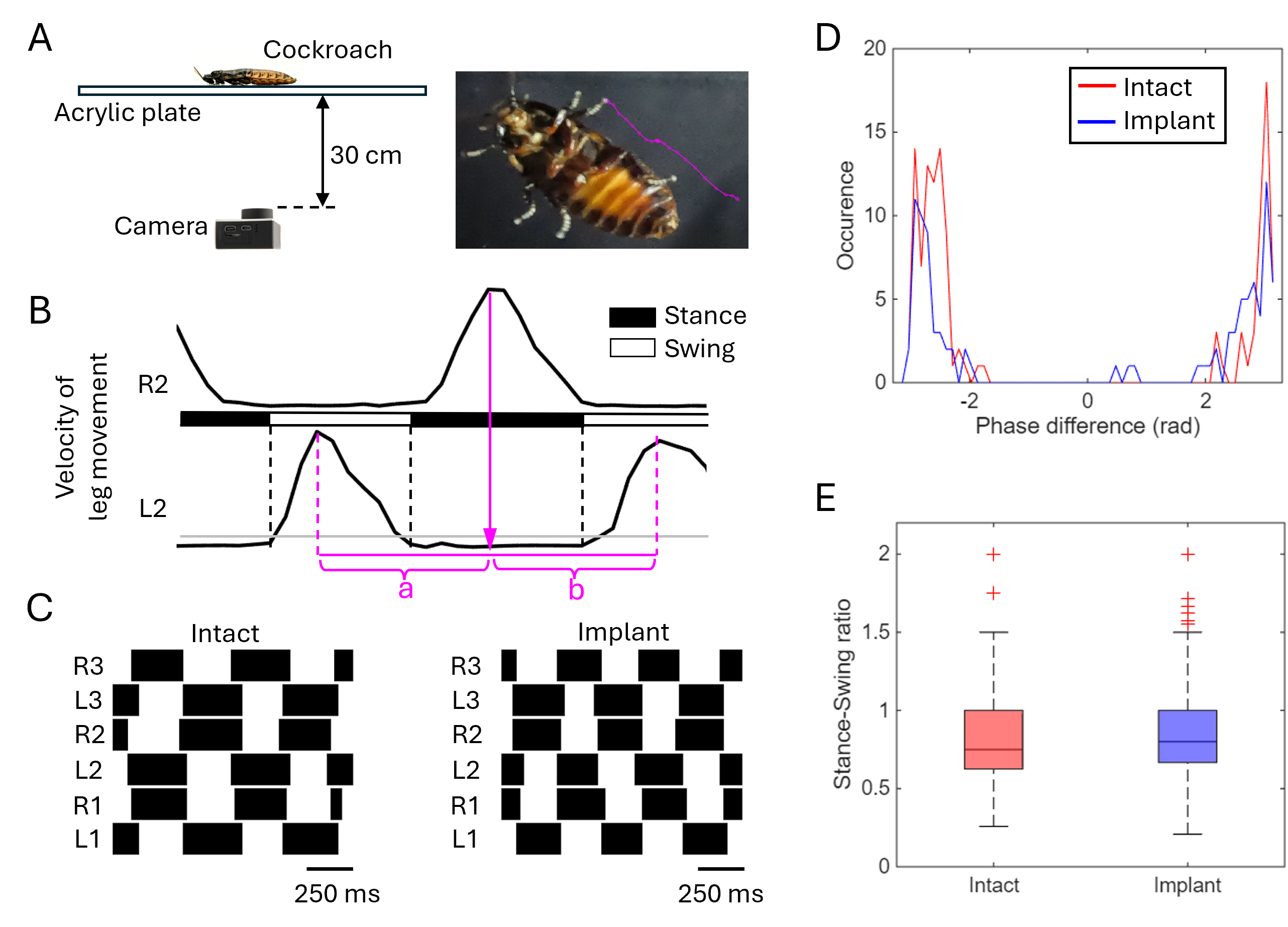

Supplement: Supplementary 1 — Figs. S1 to S5 Table S1 Movies S1 to S4 [file cbsystems.0589.f1.zip › SFig2.png]

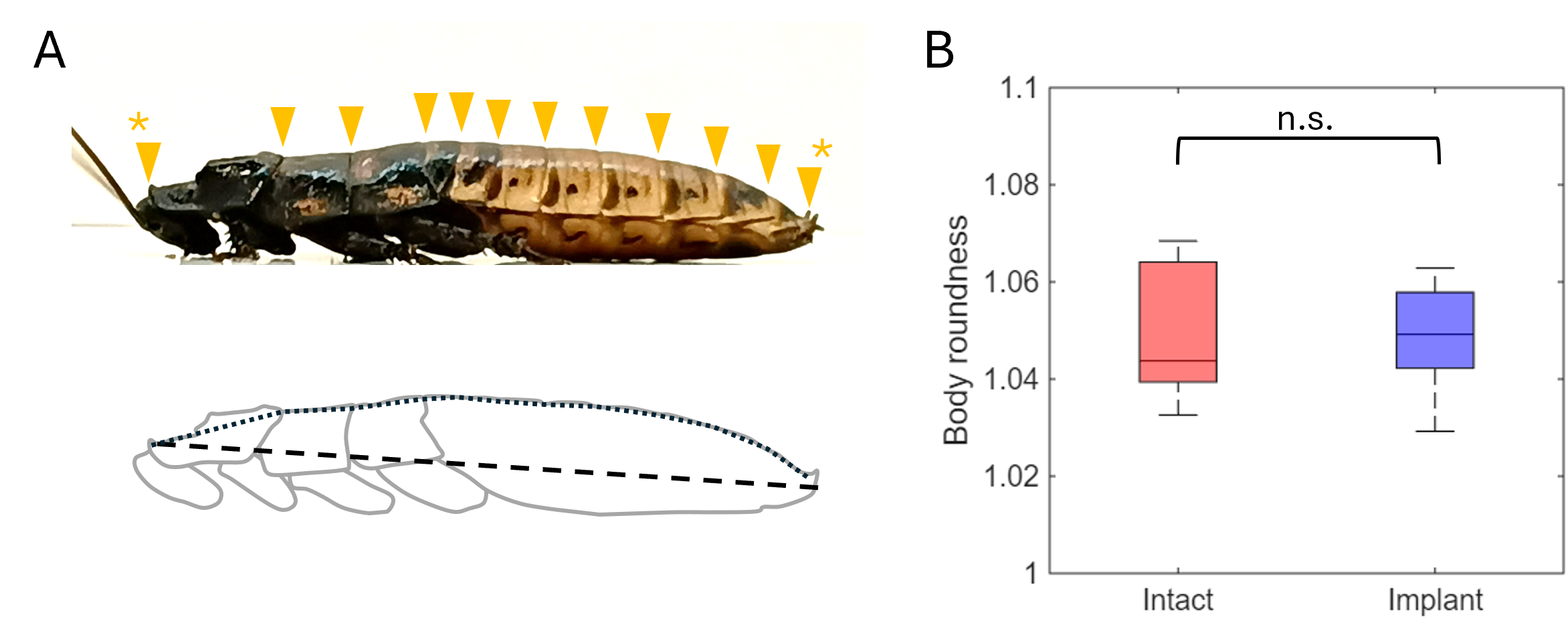

Supplement: Supplementary 1 — Figs. S1 to S5 Table S1 Movies S1 to S4 [file cbsystems.0589.f1.zip › SFig3.png]

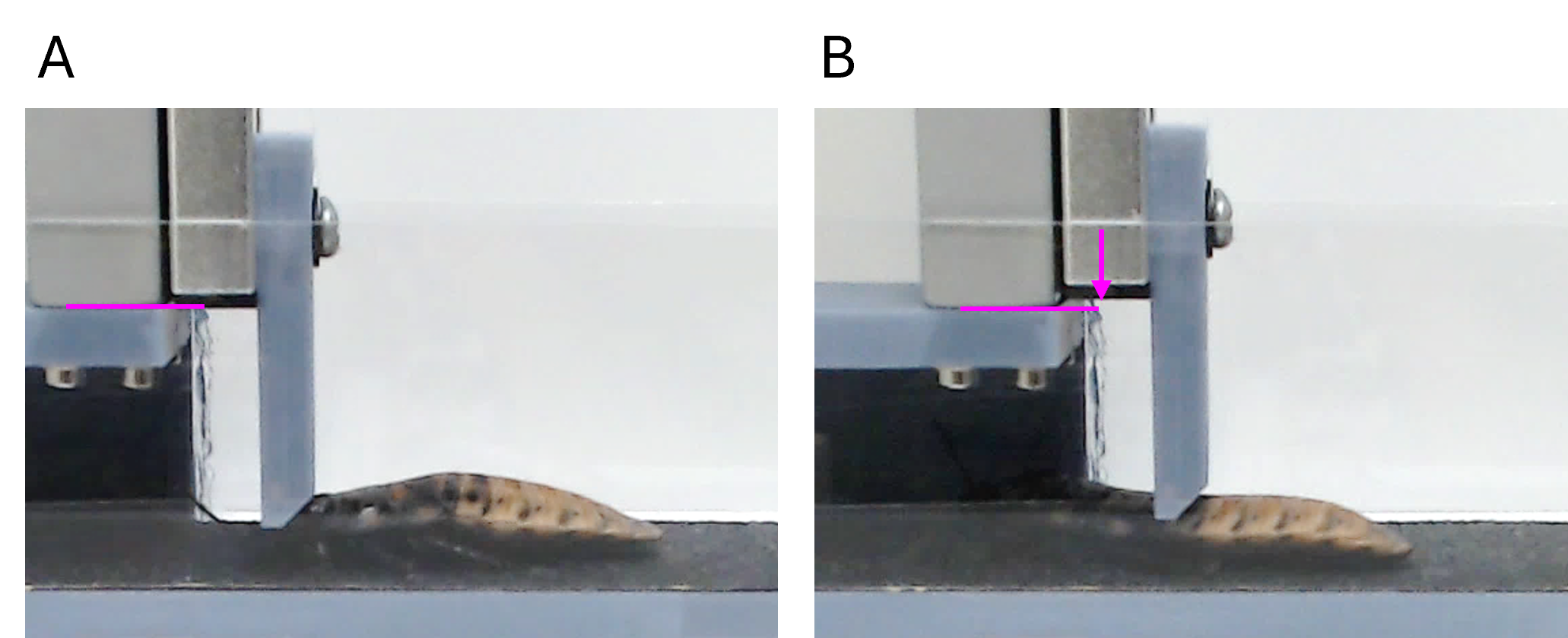

Supplement: Supplementary 1 — Figs. S1 to S5 Table S1 Movies S1 to S4 [file cbsystems.0589.f1.zip › SFig4.png]

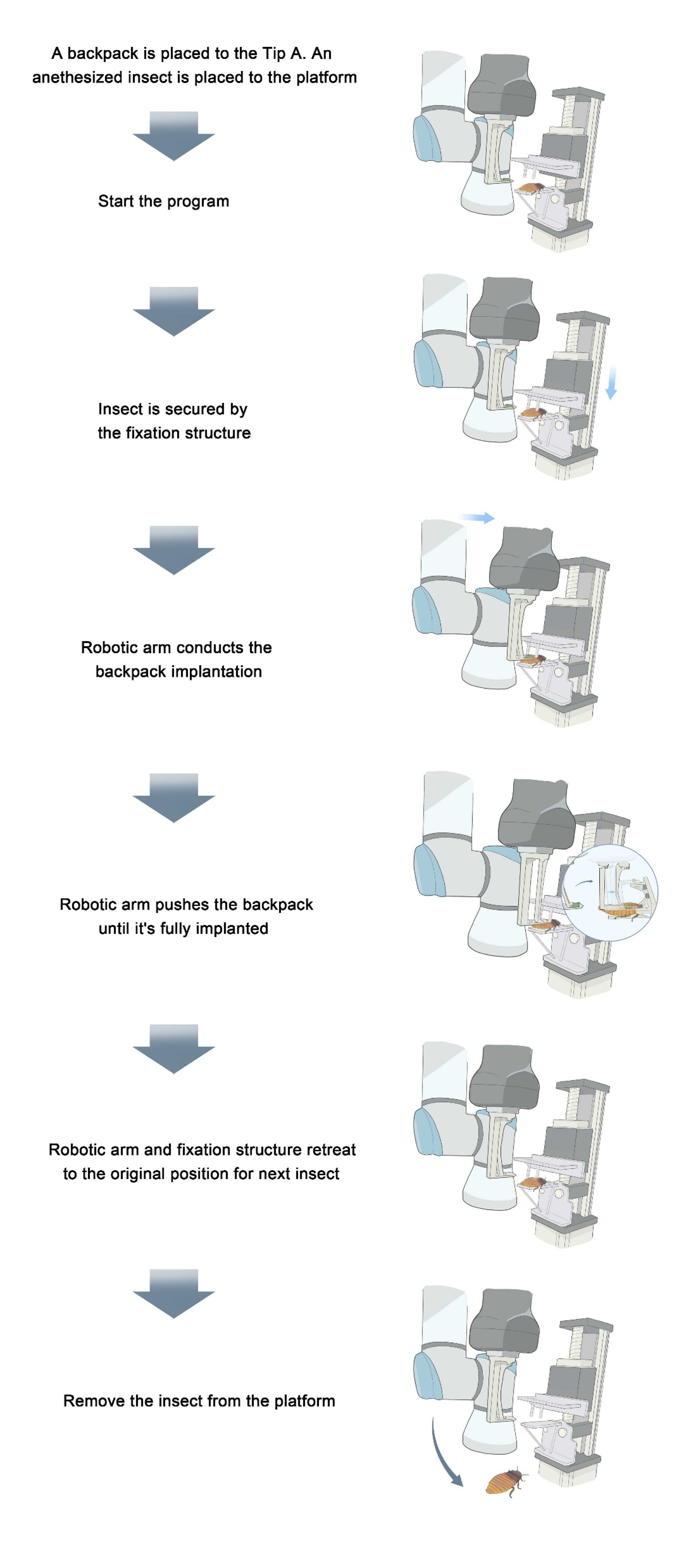

Supplement: Supplementary 1 — Figs. S1 to S5 Table S1 Movies S1 to S4 [file cbsystems.0589.f1.zip › SFig5.png]
